# Anti-Vivisectionists and the Hospital Sunday Fund

**Published:** 1909-01-02

**Authors:** George W. F. Robbins

**Affiliations:** National Anti-Vivisection Hospital, Battersea Park, S.W.


*To the Editor of* The Hospital.

### ANTI-VIVISECTIONISTS AND THE HOSPITAL SUNDAY FUND.

*To the Editor of* The Hospital.

Sir,—With reference to your excellent report of the Rev. Lionel Lewis's protest at the meeting of the constituents of the Metropolitan Hospital Sunday Fund, I think it right to point out that the sum of 3s. 3d. with which Mr. Lewis was taunted by Sir J. Bell and the Rev. C. H. Grundy as his collection for the Fund, is not the measure of that gentleman's contribution to hospital work from his poor congregation. The total amount collected was 19s. 7d., of which 16s. 4d. was specially contributed *by the donors* for this hospital, as a protest against the refusal of a grant. I have written to this effect to the Lord Mayor, Sir J. Bell, and the Rev. C. H. Grundy, and should be much obliged if you would kindly insert this letter in your next issue.

Yours faithfully,

George W. F. Robbins.

National Anti-Vivisection Hospital,
Battersea Park, S.W., December 29, 1908.

